# Timing of Intermittent Seminal HIV-1 RNA Shedding in Patients with Undetectable Plasma Viral Load under Combination Antiretroviral Therapy

**DOI:** 10.1371/journal.pone.0088922

**Published:** 2014-03-03

**Authors:** Xavier Ferraretto, Candice Estellat, Florence Damond, Pascale Longuet, Sylvie Epelboin, Pauline Demailly, Chadi Yazbeck, Marie-Astrid Llabador, Blandine Pasquet, Yazdan Yazdanpanah, Sophie Matheron, Catherine Patrat

**Affiliations:** 1 Laboratoire de Biologie De la Reproduction, Groupe Hospitalier Bichat-Claude Bernard, Paris, France; 2 Département d'Epidémiologie et Recherche Clinique, Groupe Hospitalier Bichat-Claude Bernard, Paris, France; 3 Centre d'Investigation Clinique - Epidémiologie Clinique CIE 801, INSERM, Paris, France; 4 Laboratoire de Virologie, Groupe Hospitalier Bichat-Claude Bernard, Paris, France; 5 Université Paris Diderot (VII), Paris, France; 6 Service des Maladies Infectieuses et Tropicales, Groupe Hospitalier Bichat-Claude Bernard, Paris, France; 7 Service de Gynécologie-Obstétrique, Groupe Hospitalier Bichat-Claude Bernard, Paris, France; Rush University, United States of America

## Abstract

It was demonstrated that combination antiretroviral therapy (cART) reduces the HIV-1 viral load (VL) in the blood and the seminal compartment. Some studies have reported that the seminal HIV-1 VL is undetectable in individuals with an undetectable blood plasma viral load (bpVL) under cART. However, some recent studies have demonstrated that seminal HIV-1 RNA may still be detected, and potentially infectious, even in the case of an undetectable bpVL. The aim of this retrospective study was to determine the detection rate of a seminal VL and whether shedding could be intermittent over a very short time.

From January 2006 to December 2011, 88 HIV-1 infected men, enrolled in an Assisted Reproduction program, provided 306 semen samples, corresponding to 177 frozen sperm samples (two samples delivered at a one-hour interval (n = 129) or one sample (n = 48)). All enrolled men were under cART, with an undetectable bpVL for more than 6 months. HIV-1 RNA was quantified in seminal plasma using a Roche COBAS Ampliprep COBAS TaqMan HIV-1 test.

Seminal HIV-1 RNA was detected in 23 samples (7.5%) from 17 patients (19.3%). This detection rate was stable over years. With regards to the freezing of two samples delivered at a one-hour interval, the proportion of discordance between the first and second samples was 9.3% (12/129).

Our results confirm the intermittent shedding of HIV-1 in semen. While this finding has been shown by studies examining longer time intervals, to our knowledge, this has never been demonstrated over such a short time interval.

## Introduction

HIV-1 remains wide spread throughout the population. There were approximately 6,088 new infections, resulting in a total of 150,000 infected people in France in 2011 [1]. Nearly 60% were acquired through heterosexual intercourse, despite the low efficiency of HIV-1 transmission via the sexual route (the risk of male-to-female intra-vaginal HIV-1 transmission is estimated to be approximately 8 per 1000 sexual acts) [2].

In this context, Combination Antiretroviral Therapy (cART) has remarkably improved the quality of life and the life expectancy of the HIV-1 infected population. It has also allowed serodifferent couples to consider natural procreation. Assisted Reproduction programs (AR) were established in 1992 to offer HIV-1 infected men the means to safely procreate [3]. Using AR, no seroconversion has been reported in serodifferent couples in which the men are HIV-1 infected [4].

It was demonstrated that cART reduces the HIV-1 viral load in the blood and the seminal compartment [5], [6]. Some studies have reported that the risk of HIV-1 transmission is minimal via sexual intercourse for HIV-1 infected individuals who are successfully treated with cART [7], [8], have no other sexually transmitted diseases and have had an undetectable HIV-1 plasma viral load for more than 6 months [9], [10]. However, recent studies have demonstrated that HIV-1 RNA may still be detectable and potentially infectious in semen, even if undetectable in blood plasma [11]–[15] (Table 1). The aims of this retrospective study were the following: (i) to evaluate the detection rate of a seminal viral load in a cohort of HIV-1 infected men under cART who had undetectable blood plasma HIV-1 RNA viral loads (bpVLs) for more than 6 months and (ii) to determine if HIV-1 shedding in semen could be intermittent over a very short time. We also investigated factors associated with the detection of HIV-1 RNA in seminal plasma.

**Table 1:** Summary of the different studies with seminal plasma sample analysis of HIV-1 patients under combination antiretroviral therapy and with an undetectable blood plasma viral load.

		Numbers			HIV-1 RNA detection thresholds (copies/ml)				
Studies	Studied period	Patients	SPS	Delay between bpVL and spVL	bpVL	spVL	Prevalence of detectable spVL	Prevalence of patients with at least one detectable spVL	cART regimen of patients with at least one detectable spVL**
***Vernazza et al.*** [6]	N/A	114	N/A	synchronized	400	400	N/A	1.8% (2/114)	Grp 2: 1/2, Grp 3: 1/2
***Bujan et al.*** [17]	04/1998 - 01/2001	N/A	N/A	N/A	20	100	7.9% (N/A)	N/A	N/A
***Marcelin et al.*** [12]	01/2002 - 01/2008	140*	232	synchronized	40	N/A	3% (7/232)	5% (7/140*)	Grp 1: 14.3% (1/7), Grp 2: 57.1% (4/7), Grp 3: 28.6% (2/7)
***Sheth et al.*** [11]	N/A	25	116	synchronized	50	300	16.4% (19/116)	48% (12/25)	Grp 1: 58.3% (7/12); Grp2: 42.7% (5/12)
[11] long-term follow-up	N/A	13	13	synchronized	50	30	31% (4/13)	31% (4/13)	Grp1: 2/4, Grp 2 : 2/4
***Halfon et al.*** [13]	10/2001 - 03/2009	224	263	28 days (median)	40	40	3.8% (10/263)	4% (9/224)	Grp 1: 0% (0/10), Grp 2: 50% (5/10), Grp 3: 50% (5/10)
***Dulioust et al.*** [10]	01/2002 - 12/2009	455	N/A	up to 2 months	50	100	N/A	Overall: 3.7% (17/455), 2002*: 15% (6/38), 2003*: 10% (6/60), 2004*: 6% (4/65), 2005*: 1.5% (1/80), 2006–2009 : 0% (0/212)	N/A
***Lambert-Niclot et al.*** [14]	01/2002 - 06/2011	304	628	N/A	20 or 40	100 or 200	N/A	Overall: 6.6% (20/304), 2002: 0% (0/16), 2003*: 3% (1/35), 2004*: 4% (2/30), 2005*: 3% (1/37), 2006*: 4% (1/25), 2007*: 7% (2/37), 2008*: 5% (3/60), 2009* : 6% (4/65), 2010*: 5.5% (3/55), 2011*: 11% (3/27)	Grp 1: 10% (2/20), Grp 2: 70% (14/20), Grp 3: 20% (4/20)
***Our study***	01/2006 - 12/2011	88	306	up to 6 months	50	200	Overall: 7.5% (23/306)	Overall: 19.3% (17/88)	Grp 1: 11.8% (2/17), Grp 2: 82.4% (14/17), Grp 3: 5.9% (1/17)

N/A, not available; cART, combination antiretroviral therapy; SPS, seminal plasma sample; bpVL, blood plasma viral load; spVL, seminal plasma viral load; Grp, group.

* Estimated results based on figures and numbers in published studies.

** Group 1: 1 non-nucleosidic reverse transciptase inhibitor+2 nucleosidic reverse transciptase inhibitor, Group 2: 1 protease inhibitor+2 nucleosidic reverse transciptase inhibitor, Group 3: other combination.

## Materials and Methods

We retrospectively analysed data from 88 HIV-1 infected men who enrolled in the AR program of Bichat – Claude Bernard Hospital (Paris, France), from January 2006 to December 2011. The men provided 306 semen samples, corresponding to 177 frozen sperm samples (FSs) (mean 2.0±1.4 FSs per patient). Forty-eight of the FSs were obtained from one initial sample, and 129 were obtained from two initial samples provided at a one-hour interval. The mean number of samples per patient was 3.5±2.3, with each patient providing 1 to 14 samples.

All men were under cART, with a bpVL <50 copies/ml for more than 6 months. cART regimens included two nucleosidic reverse transcriptase inhibitors (NRTIs) plus a non-NRTI or a protease inhibitor (PI) and other antiretroviral combinations. In French AR programmes, before inclusion, each couple must be validated by a multidisciplinary committee that confirms the inclusion criteria, including treatment adherence and follow-up for HIV-1 infected patients [16]. The final measure of patient blood plasma HIV-1 RNA levels was performed between 6 months before the day of FS. Patients were also asked about their treatment adherence over the past 6 months before each FS. Socio-demographic, clinical and biological data were collected at the beginning of the AR programme and included the following: age, geographic origin, sperm characteristics (semen volume, sperm concentration, mobility and vitality, seminal leukocyte counts), CD4 cell count nadir, route of HIV transmission, HBV or HCV co-infection status and cART regimen.

Semen samples were obtained by masturbation after 2–7 days of sexual abstinence. Each patient provided, if possible, 2 semen samples within a one-hour interval, according to the routine protocol in our AR centre for FS. After liquefaction, semen samples were separately centrifuged through a two-layer discontinuous gradient. After processing, the seminal plasma sample (SPS) (supernatant) was separated and transferred to the virology department for seminal plasma HIV-1 viral load (spVL) quantification using a Roche COBAS Ampliprep COBAS TaqMan HIV-1 test (Roche Diagnostics, Meylan, France) with a detection threshold of 200 copies/ml [17].

Statistical analysis was performed using SAS 9.2. The Fisher exact test and the Wilcoxon test were used, and a p-value<0.05 considered statistically significant. Our unit of analysis was the patients with at least one detectable seminal HIV-1 RNA measurement because most of the patients' characteristics used in the analysis were not repeated over time.

All samples were collected in our AR department, and all collected data were anonymised before processing. As this study included only patients referred from routine follow-up, no supplemental blood punctures or sperm retrieval were performed. Obtaining written consent is not required, as stipulated by the French Government Rule (“Loi informatique et Liberté” – Chapter IX, Article 57). The study protocol and lack of patient consent was approved by the French national commission of information and individual liberties (CNIL).

## Results

**Figure 1:**
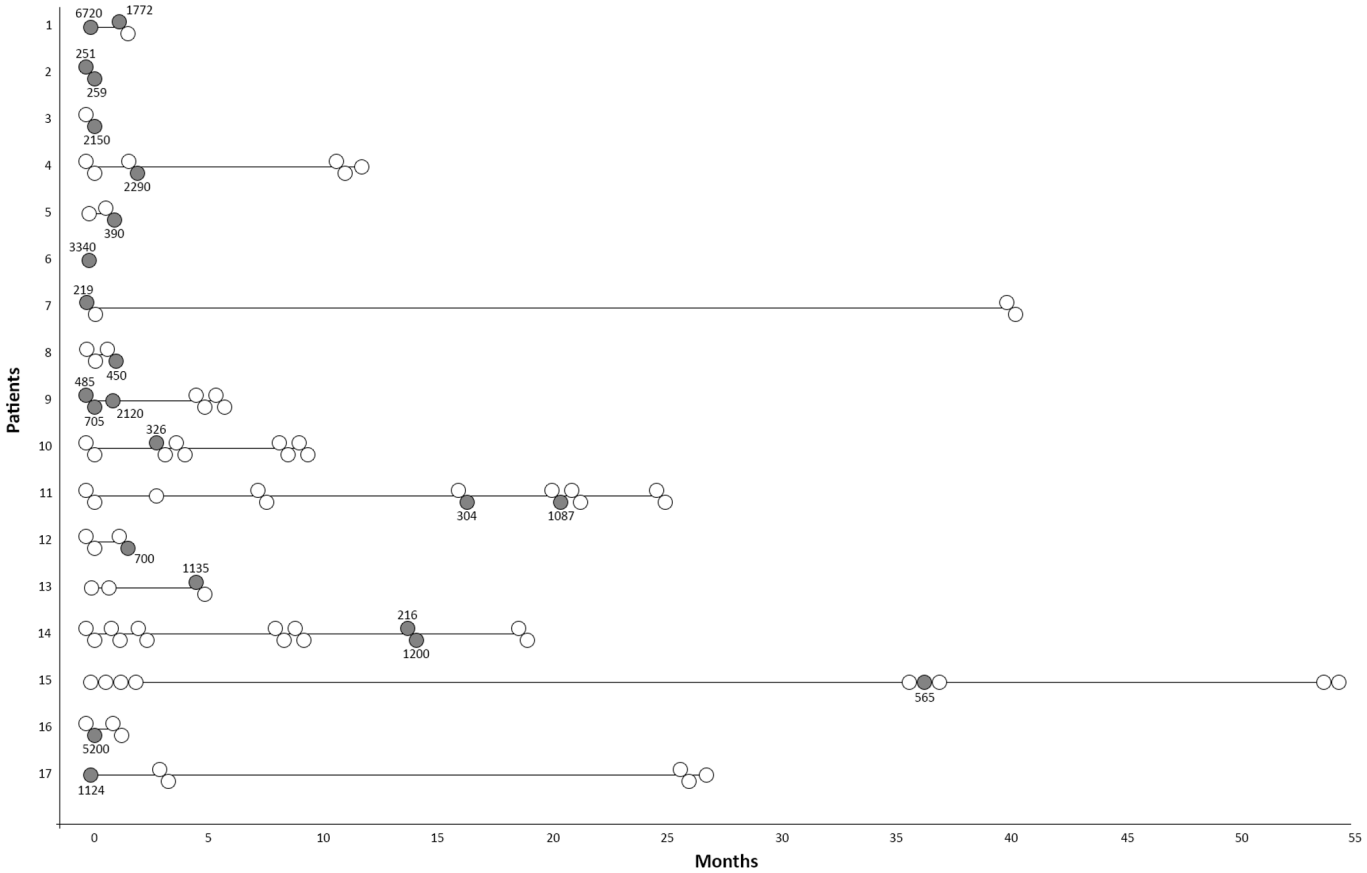
Pattern of HIV-1 shedding in the semen of 17 patients with at least one detectable seminal plasma viral load. Each horizontal line represents the data for one subject. Seminal plasma viral loads are represented by circles coloured as follows: white, undetectable; dark grey, detectable. The viral loads (copies/ml) of detectable samples are annotated near the dark grey circles. Two juxtaposed circles represent samples provided at a one-hour interval.

**Figure 2:**
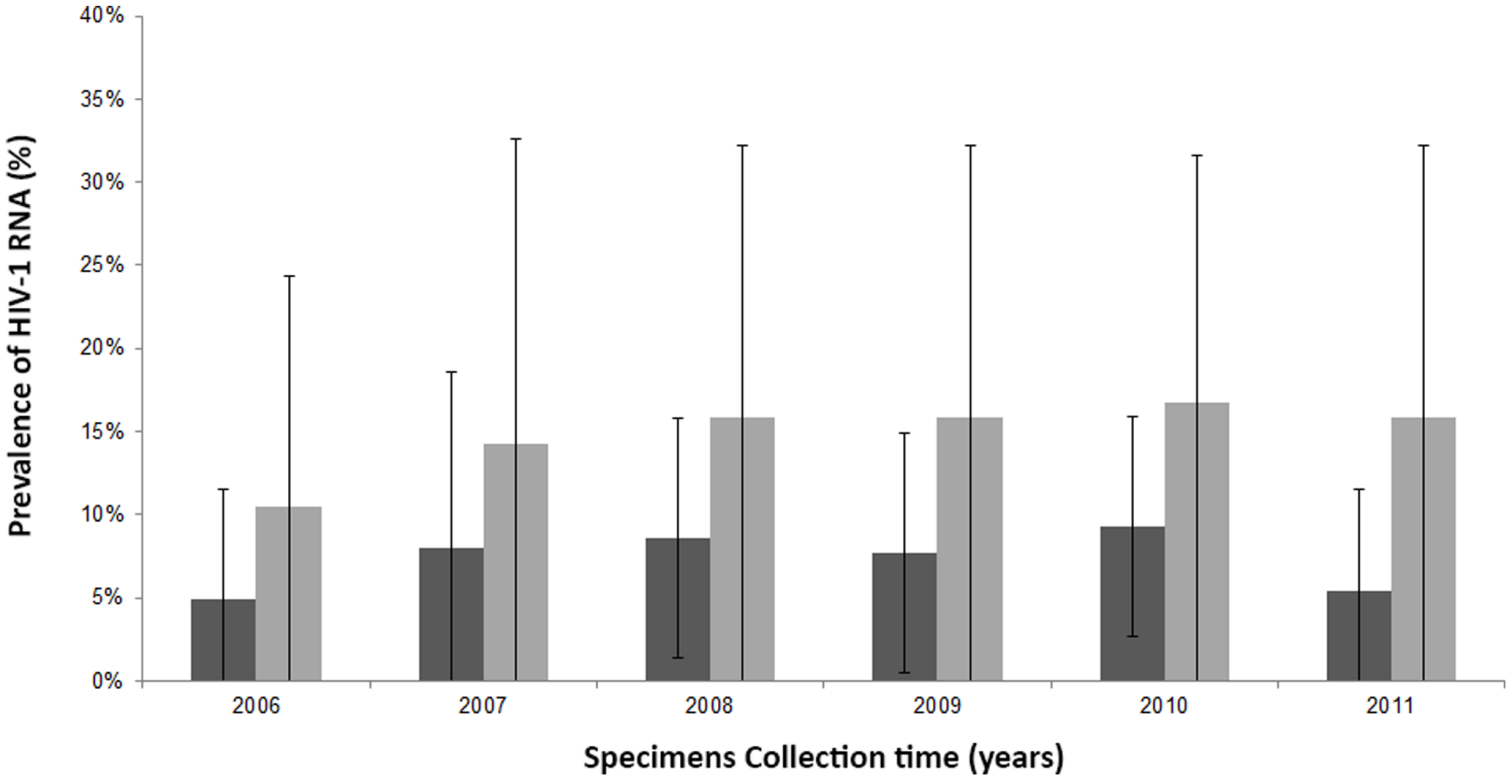
Prevalence of detectable seminal plasma samples (dark grey) and of men with at least one detectable spVL (light grey) from 2006 to 2011. Bars represent the 95% confidence intervals.

HIV RNA was detected in at least one SPS for 17 patients (19.3%) during the study period, corresponding to 23 SPSs (7.5%) (Table 2). SpVL ranged between 200 and 300 copies/ml in 4 SPSs, between 300 and 1000 copies/ml in 8, and between 1000 and 3000 copies/ml in 8; the spVL was above 3000 copies/ml in 3 SPSs (median 705 copies/ml) (Figure 1). The detection rate of spVL and the rate of patients with at least one SPS with a detectable viral load were stable over time (Figure 2, Table 1).

**Table 2:** Characteristics of the 17 patients with at least 1 detectable HIV-1 seminal plasma viral load.

Patient	Age (years)	Co-infection	HIV transmission route	CD4 cell count (cells/µl)	Antiretroviral regimen
1	31–35	/	transfusion	831	TVF, ABC, ATZ, RTV
2	31–35	/	heterosexual	302	TVF, FTC, LPV, RTV
3	41–45	/	homosexual	624	TVF, DDI, ATZ, RTV
4	41–45	HCV	heterosexual	489	TVF, FTC, EFV
5	36–40	/	heterosexual	417	TVF, FTC, EFV, LPV, RTV
6	31–35	/	heterosexual	294	ZDV, 3TC, ATZ, RTV
7	36–40	HBV	undetermined	419	TVF, FTC, ATZ, RTV
8	40–45	/	heterosexual	545	TVF, FTC, ATZ, RTV
9	46–50	HCV	drug addiction	830	DDI, 3TC, LPV, RTV
10	31–35	/	drug addiction	302	TVF, FTC, LPV, RTV
11	36–40	/	heterosexual	906	ZDV, 3TC, FPV, RTV
12	31–35	/	heterosexual	429	ZDV, 3TC, LPV, RTV
13	41–45	/	heterosexual	1676	ZDV, 3TC, ATZ, RTV
14	31–35	/	heterosexual	648	TVF, FTC, LPV, RTV
15	36–40	/	heterosexual	706	ZDV, 3TC, LPV, RTV
16	41–45	/	heterosexual	401	TVF, FTC, EFV
17	31–35	/	undetermined	609	TVF, FTC, ATZ, RTV

3TC, lamivudine; ATZ, atazanavir; ABC, abacavir; DDI, didanosine; EFV, efivarenz; FTC, emtricitabine; LPV, lopinavir; RTV, ritonavir; TVF, tenofovir; ZDV, zidovudine.

Of the 129 FSs in which two samples were provided at a one-hour interval, the spVL results in 117 (90.7%) were concordant for the first and the second SPSs, and 12 (9.3%) were discordant. In 8 cases, a spVL was undetectable in the first SPS and detectable in the second. In 4 cases, a spVL was first detectable and later undetectable. There was no significant association with the timing of the specimen. In the 12 discordant cases, the median spVL was 918 copies/ml, with a range between 200 and 500 copies/ml in 5, and between 500 and 1000 copies/ml in one; the spVL was over 1000 copies/ml in 6 cases (Figure 1).

No significant correlation between a detectable spVL and the collected data was demonstrated. More men treated with 2 NRTIs+1 PI had at least one detectable spVL (28.6%; 14/49) compared to those treated with 2 NRTIS+1 NNRTI (7.7%; 2/26) or other cART regimens (7.7%; 1/13); however, this association was not statistically significant (p = 0.054).

## Discussion

We show that HIV-1 RNA levels were above 200 copies/ml in 7.5% (23/306) of the collected seminal plasma samples from patients administered cART and with undetectable blood plasma HIV-1 RNA for at least 6 months. This rate was stable during the study period. This result is consistent with some previous studies [13], [14], different from others, which reported that the spVLs was undetectable in all samples collected after 2005 [10]. In our study, the percentage of patients with detectable HIV-1 RNA in semen (19.3%) was higher than the percentages previously reported, despite a similar or lower detection threshold (Table 2). One explanation could be the longer period (up to 6 months) between the last available blood plasma sample used for the HIV-1 RNA quantification compared with the spVL. Nevertheless, the overall detection rate of a spVL was not correlated with this delay (mean delay of 2.8±1.7 months for samples with undetectable HIV-1 RNA versus 2.6±1.7 in detectable samples).

The intermittent seminal shedding of HIV-1 RNA observed in samples collected at a one-hour interval is in agreement with that reported for longer intervals [18], [19]. Of note, this intermittence has not yet been demonstrated for such timing (one-hour intervals).

The tendency towards a higher risk of a detectable spVL in patients given the PI-containing cART regimen compared to a regimen containing an NNRTI might be explained by the poor diffusion of most PIs in the male genital tract [20]. This treatment regimen is given to 50–70% of patients with at least one sperm sample with a detectable viral load in the reported series, whereas studies describing patients with permanently undetectable HIV-1 RNA in semen occasionally report treatment regimen [11]–[14] (Table 1).

In conclusion, we show that intermittent shedding of HIV-1 RNA in the semen of patients given efficient cART could occur within a one-hour interval. This timing should not be considered to place individuals at greater risk for HIV transmission than previously reported. Indeed, the probability of HIV transmission from a sperm viral load of 1,000 copies/ml has been estimated to be 3 per 10,000 episodes according to a probabilistic empiric model [21]. The risk of heterosexual transmission of HIV-1 has been reported as non-significant (0 per 100 person-years, 95% CI = 0–0.05) when full virologic suppression on cART is achieved in the infected partner [8] and when pre-exposure prophylaxis is used for uninfected women for unprotected sexual intercourse during fertile days [22]. Nevertheless, the 7.5% detection rate of HIV-1 RNA in seminal plasma samples from the same successfully treated patients and the nearly 20% proportion of those HIV-1 infected men with at least one semen sample with a detectable viral load, which was stable over a five-year period, should balance messages on the individual risk of HIV transmission through unprotected sex as an exclusive preventive strategy in serodifferent couples with procreation desires.

## Supporting Information

Text S1 **Loi informatique et Liberté – Chapter IX, Article 57.** French government rule on treatment of personal data in medical studies.
(DOCX)
