# Supplementary material for: Timing of Intermittent Seminal HIV-1 RNA Shedding in Patients with Undetectable Plasma Viral Load under Combination Antiretroviral Therapy
Source: PLoS One. 2014 Mar 3;9(3):e88922. doi: 10.1371/journal.pone.0088922 (PMC3940424; doi:10.1371/journal.pone.0088922)
Supplement: Text S1 — Loi informatique et Liberté – Chapter IX, Article 57. French government rule on treatment of personal data in medical studies. (DOCX) [file pone.0088922.s001.docx]

**Text S1. Loi informatique et Liberté – Chapter IX, Article 57**

**Chapitre IX** - TRAITEMENTS DE DONNÉES À CARACTÈRE PERSONNEL AYANT POUR FIN LA RECHERCHE DANS LE DOMAINE DE LA SANTÉ

**Article 57**

“The individuals from whom the personal data are obtained or whose data are transmitted shall, before the start of the processing of these data, be informed individually of:

1° the nature of the transmitted information;

2° the purpose of the data processing;

3° the individuals or legal entities who are the recipients of the data;

4° the right of access and the rectification provided for in Articles 39 (right of access) and 40 (right of rectification);

5° the right to object provided for in the first (objection to the lifting of professional secrecy) and third (refusal of processing after death) paragraphs of Article 56 or, in the case provided for in the second paragraph of this Article, about the obligation to obtain their consent.

However, it is permissible for this information not to be delivered if, for legitimate reasons that the attending physician assesses honestly, a grave diagnosis or prognosis has been deliberately kept from the patient.

**Where the data have initially been obtained for a purpose other than processing, it is possible to depart from the obligation of individual information when the data subjects can no longer be located. Departure from the obligation to inform individuals about the use of data related to them for purposes of research shall be mentioned in the application for authorisation submitted to the CNIL, which decides on this point**.
